# Alcohol and Tobacco Increases Risk of High Risk HPV Infection in Head and Neck Cancer Patients: Study from North-East Region of India

**DOI:** 10.1371/journal.pone.0140700

**Published:** 2015-10-16

**Authors:** Rupesh Kumar, Avdhesh Kumar Rai, Debabrata Das, Rajjyoti Das, R. Suresh Kumar, Anupam Sarma, Shashi Sharma, Amal Chandra Kataki, Anand Ramteke

**Affiliations:** 1 Cancer Genetics and Chemoprevention Research Group, Department of Molecular Biology and Biotechnology, Tezpur University, Tezpur, Assam, India; 2 DBT center for Molecular Biology and Cancer Research, Dr. B. Borooah Cancer Institute, Guwahati, Assam, India; 3 Department of Head and Neck Oncology, Dr. B. Borooah Cancer Institute, Guwahati, Assam, India; 4 Division of Molecular Genetics, Institute of Cytology and Preventive Oncology (ICMR), I-7, Sector 39, Noida, Uttar Pradesh, India; 5 Department of Pathology, Dr. B. Borooah Cancer Institute, Guwahati, Assam, India; 6 Division of Epidemiology and Biostatistics, Institute of Cytology and Preventive Oncology (ICMR), I-7, Sector 39, Noida, Uttar Pradesh, India; 7 Department of Gynecologic oncology, Dr. B. Borooah Cancer Institute, Guwahati, Assam, India; National Health Research Institutes, TAIWAN

## Abstract

**Background:**

Human papilloma virus (HPV) associated Head and Neck Cancers (HNCs) have generated significant amount of research interest in recent times. Due to high incidence of HNCs and lack of sufficient data on high-risk HPV (hr-HPV) infection from North -East region of India, this study was conceived to investigate hr-HPV infection, its types and its association with life style habits such as tobacco, alcohol consumption etc.

**Methods:**

A total of one hundred and six primary HNC tumor biopsy specimens were collected. These samples were analyzed for hr-HPV DNA (13 HPV types) using hybrid capture 2 (HC2) assay and genotyping was done by E6 nested multiplex PCR (NMPCR).

**Results:**

The presence of hr-HPV was confirmed in 31.13% (n = 33) and 24.52% (n = 26) of the HNC patients by nested multiplex PCR (NMPCR) and HC2 assay respectively. Among hr-HPV positive cases, out of thirteen hr- HPV types analyzed, only two prevalent genotypes, HPV-16 (81.81%) followed by HPV-18 (18.18%) were found. Significant association was observed between hr-HPV infection with alcohol consumption (p <0.001) and tobacco chewing (p = 0.02) in HNC cases. Compared to HPV-18 infection the HPV-16 was found to be significantly associated with tobacco chewing (p = 0.02) habit.

**Conclusions:**

Our study demonstrated that tobacco chewing and alcohol consumption may act as risk factors for hr-HPV infection in HNCs from the North-East region of India. This was the first study from North-East India which also assessed the clinical applicability of HC2 assay in HNC patient specimens. We suggest that alcohol, tobacco and hr- HPV infection act synergistically or complement each other in the process of HNC development and progression in the present study population.

## Introduction

The Head and Neck cancer (HNC) incidence accounts for approximately 25–30% of all cancer cases in India [1]. HNC is a multifactorial and multiphasic disease which affects anatomical sites such as lip, oral cavity, nose and paranasal sinuses, nasopharynx, oropharynx, oral cavity, hypopharynx, larynx etc [2]. The crucial risk factors are tobacco, alcohol consumption, betel nut chewing, changing sexual behavior etc. which are responsible for the majority of HNC burden. In HNC patients of 50 years and above age, associations with the above key risk factors are more predominant. Tobacco smoking and alcohol related carcinogens play important role in development of HNC probably through immune suppression [3–6]. In the extracellular and intracellular compartment, cigarette smoke generates particulate matter, gaseous extracts and water solutes. Major classified mutagenic and carcinogenic components of cigarette are nicotine, tar, ammonia, carbon monoxide, carbon dioxide, formaldehyde, acrolein, acetone, benzopyrenes, hydroxyquinone, nitrogen oxides and cadmium. Tar and nicotine of the cigarette smoke affect innate immune response and increase the susceptibility to infections [7, 8]. Alcohol consumption is also considered as one of the risk factors which may contribute to carcinogenesis. The International Agency for Research on Cancer of the World Health Organization has categorized alcohol as a Group 1 carcinogen [9]. Human Papillomavirus (HPV) was advocated first time by Syrjanen et al (1983) as a risk factor especially for oropharyngeal and oral cancer [10]. In subset of HNC cases, HPV association have been acknowledged in younger age group (<50 yrs) patients without habit of tobacco and alcohol consumption [11, 12]. The HPV genomic DNA was mostly detected by PCR based method and studies have shown that up to 60% of HNC cases may be HPV positive [13, 14]. The causal association between HPV and head and neck cancer remains contradictory due to conflicting evidences [15, 16]. HPV association with HNC has assumed significance due to the findings that HPV positive HNC cases have good prognosis as compared to the HPV negative cases [17–21]. The possible routes of transmission of HPV in HNC may be oral sexual behavior in adults and perinatal transmission in the neonatal children. In particular, the oral cavity, pharynx and larynx, epithelial cells are more susceptible to HPV infection [2, 22–26]. HPV types are classified as high-risk (HPV-16, 18, 31, 33, 35, 39, 45, 51, 52, 56, 58, 59, 68, 73 and 82) and low-risk (HPV-26, 30, 34, 53, 66, 67, 69, 70, 73, 82, 85) on the basis of their carcinogenic potential. HPV-16 and HPV-18 high-risk type have been considered as major contributory genotypes in HNC [27, 28].

The master cell cycle regulators, p53, pRB and p16 are important tumor suppressor genes having significant role in cell cycle regulatory pathway and cancer [29]. These genes play important role in maintaining genomic integrity and cell cycle, and control of apoptosis [30, 31]. The High Risk HPV (hr-HPV) types 16 and 18 principally exercise their carcinogenic potential through the expression of E6 and E7 oncoprotein. The E6 oncogene activation leads to degradation of p53 through its interaction with the E3 ubiquitin ligase E6AP [31, 32]. The active E7 degrades the retinoblastoma tumor suppressor protein (pRb) due to which transcription factor E2F is stimulated and results in overexpression of p16 INK4A, a cyclin-dependent kinase inhibitor [31, 32]. By evading the above mentioned master guardians of cell cycle, HPV genes take control over the cellular proliferation that leads to uncontrolled cell division.

The North Eastern (NE) region of India has high age adjusted incidence rate (AAR) per 1, 00,000 persons for HNCs in India [33]. Among all population based cancer registries (PBCR), the Kamrup Urban district (KUD) of NE region of India was ranked second (AAR-156.3) in females and fourth (AAR-185.2) in males for all cancer sites incidence. The AAR for leading HNC sites in KUD PBCR is as follows: tongue 9.4 (male), 3.2 (female); mouth 7.7 (male), 7.6 (females); hypo pharynx 7.7 (male) and 7.6 (females); larynx 8.2(male). Tobacco related cancers (TRC) accounted for >40% but <50% in males whereas in females it was >30% but <40% of all HNC cases in KUD PBCR [33]. The population of NE region represents unique ethnicity, distinctive life style and food habits which can play important role in the complex interplay of environmental and genetic factors that may be associated with high incidence of HNC in this region. There is lack of sufficient data on the hr-HPV status in HNC cases of NE India. The aim of this study was to investigate the prevalence of hr-HPV infection and their association with betal quid chewing, smoking, tobacco, alcohol consumption and clinico-pathological characteristics of patients. In the present study, E6 nested multiplex PCR method was used for the sensitive and type-specific detection of HPV infections based on the amplification of the viral E6/E7 oncogene as described previously [28, 34]. The Hybrid Capture 2 (HC2) test, which is FDA-USA approved and WHO recommended for detection of HPV in clinical specimens of cervical intraepithelial lesions (CINs) have been applied for the first time for hr-HPV detection in HNC specimens of NE India.

## Materials and Methods

### Patients Characteristics

A total of 106 HNC patients were enrolled in this study (male 73; female 33). Tumor tissue specimens were obtained from the Head and Neck oncology surgery unit of Dr. Bhubaneswar Borooah Cancer Institute (BBCI), Regional Cancer Center, Guwahati, India. The histopathologically confirmed HNC cases were enrolled during the period of October, 2011 to September, 2013. The demographic and lifestyle information were collected using a pre-designed questionnaire through personal interview. The information collected about demographic variables included age, gender, ethnicity, education, socio-economic status, life style factors of alcohol, cigarette smoking, betel nut chewing and other food habits. The American Joint Committee on Cancer’s TNM staging was used for staging and diagnosis. The patients were informed about the study and written consent was obtained prior to collection of the specimens. This study was approved by the institutional ethics committee of Dr. Bhubaneswar Borooah Cancer Institute (BBCI), Guwahati, Assam (India).

### Genomic DNA isolation from tumor tissue

Tissue biopsy specimens were collected in 1mL volume of phosphate buffered saline (PBS) and the genomic DNA was extracted from the homogenized tissue by using QIAamp DNA mini kit (Qiagen, Germany) following the manufacturer’s instructions. The quantity and quality of the isolated genomic DNA was confirmed by Nano-spectrophotometer (Bio-photometer, Eppendorf, Germany) and agarose gel electrophoresis respectively.

### High -risk HPV DNA detection by Hybrid Capture 2 (HC2) Assay

Genomic DNA isolated from biopsy samples of the patients were suspended in the specimen transport medium (STM) (Qiagen, Germany) and the presence of HPV was detected by HC2 High-Risk HPV DNA Test^™^ kit (Qiagen, Germany) as per the instructions of the manufacturer. In the patient STM samples, chemiluminescent detection of the 13 most common hr-HPV types (16, 18, 31, 33, 35, 39, 45, 51, 52, 56, 58, 59, and 68) was performed using automated DML 2000 Luminometer system (Digene, USA). Light signals were measured as relative light units (RLUs) with light intensity indicating the presence or absence of target DNA in the tested specimen. The mean RLU value for high risk HPV calibrator of positive control samples was considered as positive cutoff (CO) value. Patients specimen with RLU/CO value ratio ≥ 1 with high-risk HPV probe were considered “positive” for any of the hr-HPV types (16,18,31,33,35,39,45,51,52,56,58,59 and 68). The absence of hr-HPV DNA below the detection limit was denoted in sample with RLU/ CO <1. When RLU = CO it confers presence of approximately 5000 virus copies in the specimen. Two confirmed cases of cervical cancer with hr-HPV positivity were also used in each test run and carrier DNA as negative control [35, 36]. The test reports were generated as per the format of the Hybrid Capture^TM^ software ver. 2.0. All the samples were tested twice and samples with hr- HPV positive outcome in both test run were only scored as hr- HPV positive.

### E6 nested multiplex PCR (NMPCR) for HPV genotyping

The samples tested positive for hr-HPV DNA in the HC2 assay were further analysed for HPV genotyping. The nested E6 PCR reaction 1^st^ round was done in 20 μL volume using 25–100 ng genomic DNA, 1X Maxima Hot Start PCR buffer, 1.5–2.0 mM MgCl_2_ (Thermo Scientific, USA), 250 nM of each primers (Metabion, Germany), 250 μM of each dNTPs and 0.5 U Maxima Hot Start Taq DNA Polymerase (Thermo Scientific, USA). The thermal cycling condition for 1^st^ round of E6 PCR was as: Denaturation- 95°C- 5 min, 38 amplification cycles with Denaturation- 95°C- 30 sec, Annealing- 55°C- 45 sec, Extension-72°C- 90 sec, and final Extension- 72°C for 7 min. The 1^st^ round E6 PCR product (630bp) was used as a template (1 μL) for the 2^nd^ round of nested reaction for HPV genotyping using multiplex set of HPV type specific primers. The reaction volume and constituents were similar as mentioned above. The 2^nd^ round nested PCR condition for HPV genotyping was similar to 1^st^ round with changes only in Annealing- 55°C- 40 sec and Extension-72°C- 45 sec [34]. All PCR products were visualized in 2% agarose gel (Amresco, USA) stained with ethidium bromide (Amresco, USA) in Gel Doc XR^™^ system (Bio-Rad, USA). The hr-HPV positive cervical squamous cell carcinoma samples DNA was used as positive control in each PCR run. The primer sequences are described in Table 1.

**Table 1:** HPV type-specific nested PCR Primer Sequences.

	Primers	Primers (5' to 3')	Amplicon size (bp)
	GP-E6-3F	Sense: GGG AGG TAC TGA AAT CGG T	630
	GP-E6-6B	Antisense: TCC TCT GAG TCG CCT AAT TGC TC	
**Multiplex primer set**	**High risk- HPV genotype**	**Primers (5' to 3')**	
**I**	16	Sense: CAC AGT TAT GCA CAG AGC TGC	457
		Antisense: CAT ATA TTC ATG CAA TGT AGG TGT A	
	18	Sense: CAC TTC ACT GCA AGA CAT AGA	322
		Antisense: GTT GTG AAA TCG TCG TTT TTC A	
	31	Sense: GAA ATT GCA TGA ACT AAG CTC G	263
		Antisense: CAC ATA TAC CTT TGT TTG TCA A	
	59	Sense: CAA AGG GGA ACT GCA AGA AAG	215
		Antisense: TAT AAC AGC GTA TCA GCA GC	
	45	Sense: GTG GAA AAG TGC ATT ACA GG	151
		Antisense: ACC TCT GTG CGT TCC AAT GT	
**II**	33	Sense: ACT ATA CAC AAC ATT GAA CTA	398
		Antisense: GTT TTT ACA CGT CAC AGT GCA	
	52	Sense: TAA GGC TGC AGT GTG TGC AG	229
		Antisense: CTA ATA GTT ATT TCA CTT AAT GGT	
	56	Sense: GTG TGC AGA GTA TGT TTA TTG	181
		Antisense: TTT CTG TCA CAA TGC AAT TGC	
	58	Sense: GTA AAG TGT GCT TAC GAT TGC	274
		Antisense: GTT GTT ACA GGT TAC ACT TGT	
**III**	35	Sense: CAA CGA GGT AGA AGA AAG CAT C	358
		Antisense: CCG ACC TGT CCA CCG TCC ACC G	
	42	Sense: CCC AAA GTA GTG GTC CCA GTT A	277
		Antisense: GAT CTT TCG TAG TGT CGC AGT G	
	43	Sense: GCA TAA TGT CTG CAC GTA GCT G	219
		Antisense: CAT GAA ACT GTA GAC AGG CCA AG	
	44	Sense: TAA ACA GTT ATA TGT AGT GTA CCG	163
		Antisense: TAT CAG CAC GTC CAG AAT TGA C	
**IV**	39	Sense: GAC GAC CAC TAC AGC AAA CC	280
		Antisense: TTA TGA AAT CTT CGT TTG CT	
	51	Sense: GAG TAT AGA CGT TAT AGC AGG	223
		Antisense: TTT CGT TAC GTT GTC GTG TAC G	
	66	Sense: TTC AGT GTA TGG GGC AAC AT	172
		Antisense: AAA CAT GAC CCG GTC CAT GC	
	68	Sense: GCA GAA GGC AAC TAC AAC GG	333
		Antisense: GTT TAC TGG TCC AGC AGT GG	

### Statistical Analysis

The data was represented as mean ± SD or in frequencies (%). The χ² test and Fisher exact test were employed to identify the association between clinico-pathological characteristics and hr-HPV status. Univariate analysis was done by calculating the odds ratios (ORs) along with 95% confidence intervals (CIs) and P ≤0.05 was considered to be the significance level. Epi-info Version 6 software was employed for the statistical analysis

## Results

### Characteristics of the Patient

In the study population (n = 106), 68.86% of patients (n = 73) were males and 31.13% were (n = 33) females. Patients’ age ranged from 25 to 80 years (Mean = 53.41 years). More than three fourths (80.18%) of the patients were from rural areas, rest (19.56%) were from semi urban and urban areas. Non-vegetarian dietary habit was reported by >92% of the patients (Table 2). The study population was grouped into either of ‘hr-HPV positive’ (n = 33) or ‘HPV negative’ (n = 73) based on the presence of hr-HPV DNA. Demographic characteristics of the patients and their dietary habits are mentioned in Table 2. The demographic factors were not significantly associated with hr-HPV infection (p>0.05). Out of the 106 cases, the anatomical sites among clinico-pathological characteristics of patients were of oral cavity 81.13% (n = 86), oropharynx 4.71% (n = 5), hypopharynx 2.83% (n = 3), larynx 8.49% (n = 9) in (Table 3). Percentages of patients diagnosed with advanced stage HNCs were stage III 24.52% (n = 26), IVA- 55.66% (n = 59) and IVB 5.66% (n = 6). Squamous cell carcinoma was predominant 85.85% (n = 91) histological type in the present study population.

**Table 2:** Demographic profiles and association with hr- HPV Positivity-.

Characteristic	Total Cases (n = 106) N (%)	hr-HPV positive (n = 33)N (%)	HPV—Negative (n = 73) N (%)	Unadjusted OR [95%CI]	P –value
**Gender**				**Univariate analysis**	
Male †	73 (68.86)	23 (69.70)	50 (68.49)	**1.0 (referent)**	
Female	33 (31.13)	10 (30.30)	23 (31.51)	0.95 [0.35–2.51]	0.90
**Betel quid**					
Non-chewers†	9 (8.18)	03 (9.10)	06(8.22)	**1.0 (referent)**	
Chewers	97 (91.82)	30 (90.90)	67 (91.78)	0.90 [0.18–4.89]	1.00
**Tobacco- chewers**					
Non-chewers†	35 (33.02)	13 (39.40)	22 (30.13)	**1.0 (referent)**	
Chewers	71 (66.98)	20 (60.60)	51 (69.87)	0.66 [0.26–1.71]	0.34
**Smoking habit**					
Non- smokers†	83 (78.30)	23 (69.69)	60 (82.19)	**1.0 (referent)**	
Smokers	23 (21.70)	10 (30.31)	13 (17.81)	2.01 [0.70–5.77]	0.14
**Alcohol intake**					
No-intake†	78 (73.58)	15 (45.45)	63 (86.30)	**1.0 (referent)**	
Intake	28 (26.42)	18(55.55)	10 (13.70)	7.56 [2.64–22.19]	<0.001*
**Both Tobacco-Alcohol habits**					
Non-Intake †	87 (82.07)	23 (69.69)	64 (87.67)	**1.0 (referent)**	
Intake	19 (17.93)	10 (30.31)	9 (12.33)	3.09 [1.00–9.65]	0.02*
**Both Smoking -Alcohol habits**					
Non- smokers†	94 (88.68)	27 (81.82)	67 (91.78)	**1.0 (referent)**	
Smokers	12 (11.32)	6 (18.18)	6 (8.21)	2.48 [0.63–9.74]	0.18
**Dietary habits**					
Vegetarian †	8 (7.54)	3 (9.09)	5 (6.49)	**1.0 (referent)**	
Non –vegetarian	98 (92.45)	30 (90.91)	68 (93.50)	0.74 [0.14–4.20]	0.70
**Place of residence**					
Urban†	7 (6.36)	1 (3.03)	6 (8.21)	**1.0 (referent)**	
Semi urban	14 (13.20)	3 (9.09)	11 (15.06)	1.64 [0.10–51.13]	1.00
Rural	85 (80.18)	29 (87.88)	56 (76.71)	3.11 [0.34–71.78]	0.42
**Age**					
≤50 years †	52(49.05)	16 (48.48)	36 (49.31)	**1.0 (referent)**	
>50 years	54(50.95)	17 (51.52)	37 (50.69)	1.03 [0.42–2.55]	0.93

* Statistically Significant; OR = odds ratio; CI = confidence interval;

^†^ Reference group for OR calculation. Since only one variable is significant that will be significant in multivariate analysis also

**Table 3:** Relationship between clinico-pathological characteristics and hr- HPV Positivity.

Characteristic	Total Cases (n = 106) N (%)	hr- HPV positive (n = 33) N (%)	hr-HPV Negative (n = 73) N (%)	Unadjusted OR [95%CI]	P –value
				***Univariate analysis***	
***Site***					
*Oral cavity* †	86 (81.13)	24 (72.72)	62 (84.93)	4.73[0.17–1.28]	1.40
*Laryngeal*	9 (8.49)	2 (6.06)	7 (9.58)	0.61[0.11–3.10]	0.55
*Hypopharyngeal*	3 (2.83)	2 (6.06)	1 (1.37)	4.64[0.40–53.1]	0.18
*Oropharyngeal*	5 (4.71)	5 (15.15)	0 (0.00)	-	<0.01*
*Nose and PNS*	3 (2.83)	0 (0.00)	3 (4.10)	-	0.24
***TNM Tumor stage***					
*II* †	15 (14.15)	3 (9.09)	12 (16.45)	**1.0 (referent)**	
*III*	26 (24.52)	6 (18.18)	20 (27.39)	1.20 [0.20–7.55]	1.00
*IVA*	59 (55.66)	24 (72.73)	35 (47.94)	2.74[0.62–13.81]	0.13
*IVB*	6 (5.66)	0 (0.00)	6 (8.21)	-	0.52
***Histology***					
*Verrucous Carcinoma* †	8 (7.54)	2 (6.06)	6 (8.21)	**1.0 (referent)**	
*Squamous Cell Carcinoma*	91 (85.85)	30 (90.91)	61 (83.56)	1.48[0.25–11.31]	1.00
*Others*	7 (6.60)	1 (3.03)	6 (8.21)	0.50[0.01–11.02]	1.00
***Morphology***					
*Well Differentiated* †	74 (69.81)	21 (63.63)	53 (72.60)	**1.0 (referent)**	
*Moderate Differentiated*	22 (20.75)	8 (24.24)	14 (19.17)	1.44[0.47–4.38]	0.47
*Poor Differentiated*	10 (9.44)	4 (12.12)	6 (8.21)	1.68[0.35–7.74]	0.38

* Statistically Significant OR = odds ratio; CI = confidence interval;

^†^ Reference group for OR calculation

### hr-HPV detection and its association with Demographic and Clinico-Pathological characteristics

The high risk HPV DNA was detected by nested multiplex PCR (NMPCR) in 31.13% (n = 33) and hybrid capture in 24.52% (n = 26) of the HNC patients. The sensitivity and negative positive predictively was found to be 100% each; the specificity and positive predictive value (PPV) was observed as 91.19% and 78.78% respectively. This test classified 93.29% of the cases correctly and the area under the curve was found as 89.50% (p<0.001) with 95%CI (81.30% - 97.80%). To confirm the biologically active HPVs, p16 expression was analyzed in both HPV positive and HPV negative cases, and over expression of p16 was found in HPV positive cases which is an indirect indicator for the presence of HPV (data not shown). In HC2 tested samples, mean RLU value was in the range of 99–3466, and the mean positive cut off (CO) value for hr-HPV was 347 (Qiagen, Germany) as per the instruction. The High risk HPV positive cases were genotyped for HPV subtypes using E6 nested multiplex PCR. The primer details used for the E6 nested multiplex PCR [29] are mentioned in the Table 1. The hr-HPV positive cases were further univariant analyzed for life style habits and significant association was found between hr-HPV positivity and alcohol consumption (OR = 7.56; 95% CI. 2.64–22.19, p <0.001) as indicated in Table 2. The alcohol drinker samples have shown to bear hr-HPV infection compared to non-drinkers. To look out for the synergistic or complementary effect between tobacco and alcohol habits, we observed significant association with hr-HPV positivity (OR = 3.09; 95% CI. 1.00–9.65, p = 0.02), but when cases of smokers and alcohol habits were together analyzed and calculated we found that there was no significant association with hr-HPV positivity. The betel nut chewers also had shown more hr-HPV infection. Among hr-HPV positive cases (n = 33), 39.40% (n = 13) patients were tobacco non-chewers and 60.60% (n = 20) were tobacco chewers. Non- smokers group 69.69% (n = 23) have more likelihood of hr-HPV infection compared to smokers 30.31% (10). Patients residing in rural areas have more hr-HPV positivity 87.88% (n = 29) compared to semi urban 9.09% (n = 3) and urban 3.03% (1%) area residents. There was no significant association found between hr-HPV infection and gender, betel quid, tobacco chewers, smoking habit, dietary habit, age and place of residence (Table 2).

Among hr-HPV positive cases (n = 33), oral cavity 72.72% (n = 24), larynx 6.06% (n = 2), hypopharynx 6.06% (n = 2) and oropharynx 15.15% (n = 5) had hr-HPV DNA (Table 3). There was significant association between hr-HPV infection and oropharyngeal tumor site (p = 0.01). Majority of the hr-HPV positive cases 90.90%, (30 of 33) were presented with advanced stage III, IVA or IVB disease. Histologically, 90.90% hr-HPV positive cases were squamous cell carcinoma, 6.06% were verrucous carcinoma and 3.03% were other carcinomas (adenoid cystic carcinoma, spindle cell malignant) (Table 3). There was no significant association of TNM tumor stage, histology grade and morphology with hr- HPV positivity (p>0.05). When HNC cases were analyzed with lifestyle factors, alcohol consumption was found to be significantly associated with hr-HPV infection (p<0.001) (Table 2). Next we analysed hr-HPV status in oral cavity cancers alone (Table 4) and compared with smoking, tobacco, alcohol, betel nut use and found that there was significant association of hr-HPV positivity and alcohol consumption (OR = 2.98; 95% CI. 0.95–9.43, p = 0.03). To find the synergistic or complementary effect of tobacco and alcohol habits, from univariate analysis it was found that there is significant association of hr-HPV positivity with tobacco and alcohol habits(OR = 2.94; 95% CI. 0.95–8.88, p = 0.05) (Table 4). But when analyzed the case of smokers and alcohol habits together, no significant association was found with hr-HPV positivity.

**Table 4:** Relationship of betel nut, tobacco, smoking, and alcohol and status of hr-HPV in Oral cavity patients only.

Characteristic	Oral cavity Cases (n = 86) N (%)	hr-HPV positive (n = 24)N (%)	HPV—Negative (n = 62) N (%)	Unadjusted OR [95% CI]	P –value
				***Univariate analysis***	
***Betel quid***					
*Non-chewers* †	9 (10.46)	6 (25)	3 (4.84)	**1.0 (referent)**	
*Chewers*	77 (89.54)	18 (75)	59 (95.16)	0.15[0.03–0.79]	0.01
***Tobacco- chewers***					
*Non-chewers* †	30 (34.88)	10 (41.67)	20 (32.25)	**1.0 (referent)**	
*Chewers*	56 (65.12)	14 (58.33)	42 (67.75)	0.67[0.23–1.96]	0.41
***Smoking habit***					
*Non- smokers* †	66 (76.74)	20 (83.33)	46 (74.19)	**1.0 (referent)**	
*Smokers*	20 (23.26)	4 (16.67)	16 (25.81)	0.57[0.14–2.17]	0.36
***Alcohol intake***					
*No-intake* †	64 (74.42)	14 (58.33)	50(80.64)	**1.0 (referent)**	
*Intake*	22 (25.58)	10 (41.67)	12 (19.36)	2.98[0.95–9.43]	0.03*
***Both Tobacco-Alcohol habits***					
*Non-Intake* †	69 (80.23)	16 (66.67)	53 (85.48)	**1.0 (referent)**	
*Intake*	17 (19.77)	8 (33.33)	9 (14.52)	2.94 [0.97–8.88]	0.05*
***Both Smoking -Alcohol habits***					
*Non- smokers* †	77 (89.54)	20 (83.33)	57 (91.93)	**1.0 (referent)**	
*Smokers*	9 (10.46)	4 (16.66)	5 (8.06)	2.28 [0.55–9.33]	0.25

* Statistically Significant OR = odds ratio; CI = confidence interval;

^†^ Reference group for OR calculation

### Association of HPV types 16 and 18 with Demographic and Clinico-pathological characteristics

Among hr- HPV positive cases, HPV-16 was present in 81.81% (n = 27) and HPV-18 in 18.18% (n = 6) of the cases. The tobacco chewers had significantly increased risk of HPV-16 infection (p = 0.02) but not of HPV-18. The risk of HPV-16 being positive is 11.88 times more in chewers as compared to non-chewers with 95% CI (1.01–317.2). However the smoking habit does not show any significance compared to non-smokers (OR- 0.14) with 95% CI (0.01–1.28). The HPV-18 positivity was found in 5 cases (83.33%) of non-chewers compared to chewers. HPV-16 infection in smokers was found to be higher (p = 0.05) compared to HPV-18 (Table 5), Though hr-HPV DNA was found to be significantly associated with alcohol consumption (Table 2) but with reference to HPV -16 alone or, HPV type 18 alone, there was no such association found with it. The oral cavity 70.37% (n = 19), larynx 7.41% (n = 2), hypopharynx 7.41% (n = 2) and oropharynx 14.82% (n = 4) cancer patients have HPV-16 as most predominant hr-HPV type whereas HPV -18 was found only in oral cavity 83.33% (n = 5) and oropharynx 16.66% (n = 1). In the histological subtype squamous cell carcinoma, the HPV-16 was found in 25 cases (92.59%) and HPV-18 in 5 cases (83.34%) was detected. HPV-16 was predominant in males 66.66% (n = 18) as well as proportionately high in females 33.44% (n = 9), whereas HPV-18 was more in males 83.33% (n = 5) compared to females 16.67% (n = 1) cases. Betel quid chewing, alcohol consumption, age, gender did not show any significant association with the presence of HPV-16, 18 types (p>0.05) (Table 5).

**Table 5:** Relation of HPV -16 and HPV-18 with clinico-pathological characteristics.

Characteristic	Total HPV positive (n = 33) N (%)	HPV-16 Positive *(n = 27)* N (%)	HPV-18 Positive *(n = 6)* N (%)	Unadjusted OR [95%CI]	P- value
				***Univariate analysis***	
***Gender***					
*Male* †	23 (69.69)	18 (66.66)	5 (83.33)	**1.0 (referent)**	
*Female*	10 (30.30)	9 (33.44)	1 (16.67)	2.5[0.21–15.52]	0.64
***Age***					
*≤50 years* †	16 (48.48)	12 (44.44)	4 (66.67)	**1.0 (referent)**	
*>50 years*	17 (51.51)	15 (55.56)	2 (33.33)	2.50 [0.30–24.23]	0.40
***Betel quid***					
*Non-chewers* †	3 (9.09)	1 (3.70)	2 (33.34)	**1.0 (referent)**	
*Chewers*	30 (90.91)	26 (96.29)	4 (66.66)	13.0[0.66–483.12]	0.08
***Tobacco- chewers***					
*Non-chewers* †	13 (39.39)	8 (29.63)	5 (83.33)	**1.0 (referent)**	
*Chewers*	20 (60.60)	19 (70.37)	1 (16.67)	11.88[1.01–317.21]	0.02*
***Smoking habit***					
*Non- smokers* †	23 (69.69)	21(77.78)	2 (33.33)	**1.0 (referent)**	
*Smokers*	10 (30.30)	6 (22.22)	4 (66.66)	0.14 [0.01- 1.28]	0.05*
***Alcohol intake***					
*No-intake* †	15 (45.45)	13(48.15)	2 (33.33)	**1.0 (referent)**	
*Intake*	18 (54.54)	14 (51.85)	4 (66.66)	0.54[0.06–4.43]	0.51
***Sites***					
*Oral cavity* †	24 (72.72)	19 (70.37)	5 (83.33)	**1.0 (referent)**	
*Laryngeal*	2 (6.06)	2 (7.41)	0 (0.00)	-	0.48
*Hypopharyngeal*	2 (6.06)	2 (7.41)	0 (0.00)	-	0.48
*oropharyngeal*	5 (15.15)	4 (14.82)	1 (16.66)	1.05[0.07–30.65]	0.96
***TNM Tumor stage***					
II †	3 (9.09)	3 (11.11)	0 (0.00)	-	
III	6 (18.18)	4 (14.81)	2 (33.33)	-	0.44
IVA	24 (72.72)	20 (74.08)	4 (67.67)	-	
**Histology**					
*Squamous Cell Carcinoma* †	30 (90.90)	25 (92.59)	5 (83.34)	**1.0 (referent)**	
*Verrucous Carcinoma*	2 (6.06)	1 (3.70)	1 (16.66)	0.20[0.01–8.93]	0.24
*Adenoid Cystic Carcinoma*	1 (3.03)	1 (3.70)	0 (0.00)	0.64[0.02–18.0]	0.65
***Morphology***					
*Well Differentiated* †	21 (63.63)	18 (66.67)	3 (50)	**1.0 (referent)**	
*Moderate Differentiated*	8 (24.24)	6 (22.22)	2 (33.33)	0.50[0.05–5.69]	0.49
*Poor Differentiated*	4 (12.12)	3 (11.11)	1 (16.66)	0.50[0.02–17.07]	0.59

* Statistically Significant, OR = odds ratio; CI = confidence interval;

^†^ Reference group for OR calculation

## Discussion

The Human papilloma virus (HPV) has been identified as an etiological factor in subset of head and neck cancer especially oropharyngeal cancer [37]. Most of the HPV positive associated oropharyngeal carcinoma patients were reported to be younger in age and mostly had no history of tobacco/alcohol use [38]. Some research groups have advocated that HPV positive and HPV negative HNC cases should be considered as two distinct clinical, pathological entities and the treatment regimens need to be devised accordingly [37–39]. The tumor hypoxia fraction and proliferation abilities were significantly decreased in HPV positive tumor cells after irradiation in comparison to HPV negative cells [40]. It was demonstrated that HPV positive locally advanced HNC patients have shown significantly higher pathological complete response as compared to HPV negative cases undergoing chemotherapy regimen [41]. Numerous studies have reported HPV DNA detection in HNSCC with varying rates, the detection techniques included in situ hybridization and southern blot hybridization (lower sensitivity methods) or PCR (high sensitivity methods) [42]. Moreover, methods used for sampling and storage also vary. The lack of universally acceptable standardized and clinically relevant procedure for HPV detection has raised concerns about their application in routine clinical setting. Our study has, for the first time assessed the applicability of HC2 assay in clinical specimens of HNC cases from NE India. We propose that Hybrid Capture 2 (HC2) assay, which is approved by Food and Drug Administration (FDA, USA), for hr-HPV detection in clinical cervical specimens, can also be used for hr-HPV detection in HNC specimens. Due to semi-quantitative nature of HC2 assay it may have application for prognostic assessment of hr-HPV in HNC patients.

We used E6 nested multiplex PCR (NMPCR) for HPV genotyping, and found HPV-16 in 81.81% (n = 27) and HPV-18 in 18.18% (n = 6) of the hr-HPV positive (n = 33) cases, which is 31.13% of HNC patients (33/106). Our study data is supported by previous studies from India which had found HPV DNA in the range of 22% to 73% in oral cancer cases [43–45]. Among (n = 33) hr- HPV cases, we found (n = 27) HPV 16 in which 70.37%, 14.82%, 7.41%, 7.41% and (n = 7) HPV-18 in 83.33%, 16.66%, 0%, and 0% of oral cavity, oropharyngeal, laryngeal and hypopharyngeal cancer patients respectively. Our study shows higher HPV-16, 18 infection rate compared to earlier studies from India [43–46]. We found that oropharyngeal cancer site compared to other tumor sites has shown significant risk for high-risk HPV infection. Our data is supported by earlier study, in which it was found to have association of oropharyngeal cancer with high- risk HPV infection [47]. Epidemiology of HPV positive oropharyngeal squamous cell carcinoma (OPSCC) are distinct from HPV negative ones and are characterized by younger age and strongly associated with sexual behavior. HPV associated oropharyngeal cancer (HPV-OPC) is growing in incidence and has distinct clinical, pathological, molecular and epidemiologic features [38]. The difference in our observations with other studies can be attributed to different methodologies, ethnic variation, tumor sites etc [48]. Our data show that betel nut chewing, smoking may not have significant association with hr-HPV infection. The hr-HPV positivity in the HNC cases was small which may confound the role of betel nut, smoking as risk factors for HPV infection. It was demonstrated by Ang et al (2010) that HPV positive oropharyngeal cancer progression may not be strongly associated with tobacco-smoking [47]. But other studies have shown tobacco association with HPV infection [49].

We observed in our data that tobacco chewers have significantly higher hr-HPV infection, posing tobacco chewing as a risk factor for hr- HPV infection. The concentration of nicotine and its exposure duration to the cell may alter the antigen mediated signaling pathways [50]. Alteration in the antigen mediated signaling events in the immune cells may be the mechanism by which nicotine or other constituents of cigarette inhibit the response of cell for bacterial/ viral infection [50]. We suggest that tobacco associated carcinogens may induce alterations in genetic events which may lead to molecular changes, making the individual susceptible to hr-HPV infection.

In the present study hr-HPV infection was found to be significantly associated with alcohol consumption in HNC cases. Oh et al (2014) recently demonstrated that hr-HPV load and alcohol consumption may have synergistic effect on hr-HPV infection and its persistence in cervical cancer [51]. Another recent study has shown that alcohol intake was associated with significantly increased risks for HPV infection in men [52]. This supports our observation that alcohol intake, may act as a risk factor for HPV infection.

Ethanol, a major constituent of alcoholic beverages, is oxidized to acetaldehyde by alcohol dehydrogenases (ADH). Acetaldehyde is metabolized to acetate by aldehyde dehydrogenases (ALDH). Poor oral hygiene and smoking can change the oral bacterial flora which can lead to increased acetaldehyde production [53]. In animal models, acetaldehyde has been proved as carcinogenic and mutagenic [54]. Genetic factors such as ADH/ALDH2 polymorphism, lack of ALDH2 or low levels of expression of ALDH2 and alcohol drinking habits were reported to be associated with higher risk of head and neck cancer [55, 56].

Alcohol can also modify innate immune response in dose dependent manner which can result in altered inflammatory response to infection [57]. Since immune response plays major role in HPV infection, alcohol consumption may help in evading the immune response for HPV infection. Systemic immunity components such as cytokine serum level have been found to be increased in persistent HPV infection cases [58].

Our study provides evidence about increase in vulnerability for hr-HPV infection in tobacco chewers and alcohol drinkers mostly in oral cavity cancer patients. Our observation is well supported by previous HNC studies from India and worldwide [14]. From the total number of cases when tobacco chewing and smoking habits were analyzed with HPV, the associations were not significant (Table 2), as major proportion of population were HPV negative and HPV positive cases were less. But when the chewing habits were taken along HPV positive cases alone, significant association was found (Table 4) proving that tobacco chewing and alcohol consumption act as risk factors for HPV infection. More number of oropharyngeal cancers in our study might have brought a better assessment of alcohol and tobacco association with hr-HPV in these cancers. We focused primarily on hr- HPV DNA detection by employing newer methodology such as HC2 assay and E6-NMPCR which are less used in HNC. The alcohol drinking and tobacco chewing/smoking are widespread habits in North-East Indian population. We feel that mass sensitization through screening cum awareness programmes about HNC risk factors can help in reducing the HNC incidence in the NE population in future.

We conclude from our present study data that hr-HPV infection may be more prevalent in tobacco chewers, alcohol drinkers and they act as risk factors for HPV infection in HNC cases of North-East India. Hybrid Capture 2 (HC2) assay due to its high negative predictive value, specificity and sensitivity can be applied for hr- HPV detection in HNC clinical specimens.
